# RGD Peptides-Conjugated Pluronic Triblock Copolymers Encapsulated with AP-2α Expression Plasmid for Targeting Gastric Cancer Therapy *in Vitro* and *in Vivo*

**DOI:** 10.3390/ijms160716263

**Published:** 2015-07-17

**Authors:** Wei Wang, Zhimin Liu, Peng Sun, Cheng Fang, Hongwei Fang, Yueming Wang, Jiajia Ji, Jun Chen

**Affiliations:** 1 Department of Gastric and Pancreatic Surgery, Sun Yat-sen University Cancer Center, State Key Laboratory of Oncology in South China, Guangzhou 510060, China; E-Mails: liuzhim2@sysucc.org.cn (Z.L.); fangcheng@sysucc.org.cn (C.F.); 2 Collaborative Innovation Center for Cancer Medicine, Guangzhou 510060, China; E-Mail: sunpeng@sysucc.org.cn; 3 Department of Anesthesiology, the First Affiliated Hospital of Bengbu Medical College, Anhui 233004, China; E-Mail: hongwei_fang163@163.com; 4 Department of Anatomy, Histology and Embryology, School of Medicine, Shanghai Jiao Tong University, Shanghai 200025, China; E-Mail: wangyueminghao@126.com; 5 Department of Orthopedic Sports Medicine, Huashan Hospital, Fudan University, Shanghai 200040, China; E-Mail: jijiajia1999@163.com

**Keywords:** gastric cancer, gene therapy, triblock copolymers, AP-2α, RGD

## Abstract

Gastric cancer, a high-risk malignancy, is a genetic disease developing from a cooperation of multiple gene mutations and a multistep process. Gene therapy is a novel treatment method for treating gastric cancer. Here, we developed a novel Arg-Gly-Asp (RGD) peptides conjugated copolymers nanoparticles-based gene delivery system in order to actively targeting inhibit the growth of gastric cancer cells. These transcription factor (AP-2α) expression plasmids were also encapsulated into pluronic triblock copolymers nanoparticles which was constituted of poly(ethylene glycol)-block-poly(propylene glycol)-block-poly(ethylene glycol) (PEO-block-PPO-block-PEO, P123). The size, morphology and composition of prepared nanocomposites were further characterized by nuclear magnetic resonance (NMR), transmission electron microscopy (TEM) and dynamic light scattering (DLS). In MTT (3-(4,5-dimethyl-2-thiazolyl)-2,5-diphenyltetrazolium bromide) analysis, these nanocomposites have minor effects on the proliferation of GES-1 cells but significantly decreased the viability of MGC-803, suggesting they own low cytotoxicity but good antitumor activity. The following *in vivo* evaluation experiments confirmed that these nanocomposites could prevent the growth of gastric cancer cells in the tumor xenograft mice model. In conclusion, these unique RGD peptides conjugated P123 encapsulated AP-2α nanocomposites could selectively and continually kill gastric cancer cells by over-expression of AP-2α *in vitro* and *in vivo*; this exhibits huge promising applications in clinical gastric cancer therapy.

## 1. Introduction

Gastric cancer, a high-risk malignancy, is a genetic disease developing from a cooperation of multiple gene mutations and a multistep process [1,2,3]. Gene therapy as a novel treatment method has achieved improvements over past decades, particularly for treating malignant cancer [4,5]. However, there are still some unsolved problems, limiting the further development of gene therapy for malignant cancer. Thus, it is crucial to choose an appropriate target therapy gene such that the effects of gene therapy are limited to cancer cells. Recently, several preliminary studies of targeted genes in gastric cancer have been performed [6,7,8]. However, a lack of suitable target genes is still the major obstacle for gene therapy of gastric cancer.

Activating protein 2 family (AP-2) is a sequence-specific DNA-binding transcription factor [9]. These factors are required for normal growth and morphogenesis during mammalian development and are expressed in neural crest lineages and regulated by retinoic acid [10,11]. Recent studies have shown that AP-2α can regulate cell function by targeting related cancer genes such as the ErbB2 proto-oncogene, the cell-cycle control gene cyclin-dependent kinase inhibitor 1A (*CDKNI1A*), and the cell adhesion and invasion genes matrix metallopeptidase 2 (*MMP-2*), matrix metalloproteinase 9 (*MMP-9*) and pseudoautosomal-1 (*PAR-1*) [12]. The over-expression of the AP-2α protein can inhibit the expression of Bcl-2 protein, stimulate the bax/cytochrome/Apaf1/caspase-9 signaling pathways, and finally induce apoptosis in tumor cell. In addition, the AP-2α protein can enhance the efficacy of chemotherapy drugs against tumors [13,14]. Previous studies not only observed a clear inhibition of tumor growth in mice pancreatic cancer tumor model, but also found that the efficacy of gemcitabine is enhanced by up-regulating the expression of AP-2α protein [15]. Based on our robust previous studies, 41 pairs of primary gastric cancer tissues including tumor tissues and tumor-adjacent tissues were selected to investigate the differences in expression of AP-2α protein by real time-PCR and Western blotting. The results showed that the expression of AP-2α in tumor tissue was dramatically lower than that in tumor-adjacent tissue [16]. Moreover, after up-regulating AP-2α expression in gastric cancer cells by transfection, our study revealed that these gastric cancer cells’ growth could be inhibited, and finally result in cell apoptosis (data not shown) [17].

In previous research [18], the lentivirus was normally employed to regulate the expression of target gene. However, the major drawback for lentivirus, as one of the viral vectors, is the lack of specificity, which increases the risk of inadvertent germline gene transfer after gene therapy, and also raises additional safety and ethical concerns. The rapid development of nanotechnology provides promising approaches for designing delivery system. Polymer micelle is one of the most promising nanoscale delivery system because of their good biocompatibility and biosafety. Furthermore, they also exhibit a prolonged circulation time in the blood owing to their evading scavenging ability by the mononuclear phagocyte system (MPS) [19], avoiding the reticuloendothelial system (RES) and renal clearance stem from the hydrophilic micelles shell. Therefore, polymer micelles are considered to be an excellent delivery system for gene therapy. So far, some natural or synthetic block copolymers have been employed to form polymeric micelle delivery system based on poly(ethyleneoxide) (PEO) as the water-soluble block [20,21,22]. Among them, poly(ethyleneglycol)*_x_*-block-poly(propylene glycol)*_y_*-block-poly(ethylene glycol)*_x_*(PEO*_x_*-block-PPO*_y_*-block-PEO*_x_*, known commercially as Pluronics or Poloxamers) triblock copolymers have been commonly used for solubilization of hydrophobic anti-neoplastic drugs in a variety of molecular weights and EO/PO ratios [23]. Since 2010, our group has developed a novel carboxyl-functionalized Pluronic P123 triblock copolymers [24]. Herein, we are aim to evaluate the feasibility of using those RGD peptides conjugated Pluronic P123 triblock copolymers as a novel delivery *AP-2α* gene expression plasmid nanosystem (RGD@P123@AP-2α) instead of lentivirus for gastric cancer treatment.

## 2. Results and Discussion

### 2.1. Characterization of RGD@P123@AP-2α Nanocomposites 

**Figure 1:**
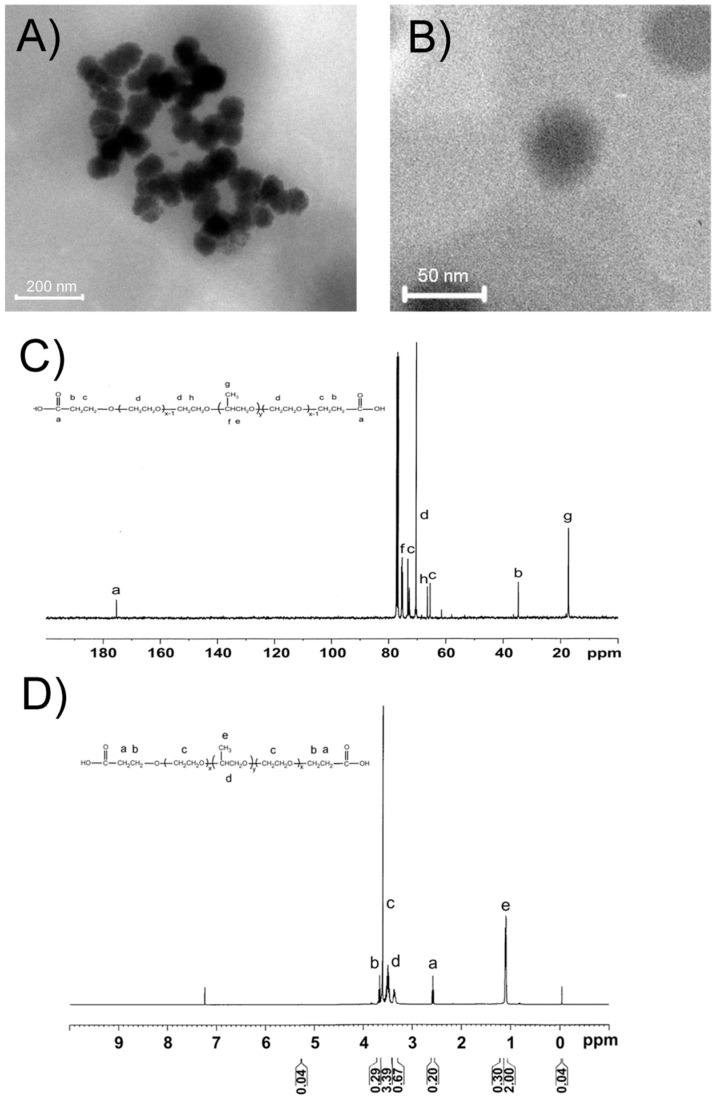
(**A**) Transmission electron microscopy (TEM) and (**B**) High resolution (HR)-TEM images of as-prepared RGD@P123@AP-2α nanocomposites; The (**C**) ^13^C and (**D**) ^1^H NMR spectrum of P123.

As shown in Figure 1A, all of freshly prepared RGD@P123@AP-2α nanocomposites were spherical in shape, and have coarse surface, whereas the average diameter is about 50 nm, which was confirmed by the high-resolution TEM image (HR-TEM) (Figure 1B). Furthermore, the ^13^C and ^1^H NMR spectrum of P123 were also shown in Figure 1C,D, respectively. According to the ^13^C NMR spectrum in Figure 1C, the signal at 17.30, 18.74, 61.6, 65.79, 70.50, 72.80, 75.0 and 175.4 ppm are assigned to –CH_3_, –CH_2_–, –CH_2_–OH, –CN, EO–CH_2_–CH_2_–, PO–CH_2_–, PO–CH– and –COOH groups, respectively. As a supplementary, the ^1^H NMR gives a further detailed formation conform of P123. The mean diameter and particle size distribution of these nanocomposites were determined by dynamic light scattering (DLS) measurement. As shown in Table 1, the mean sizes of blank copolymer P123, P123@AP-2α and RGD@P123@AP-2α were 19.3 ± 1.5, 21.2 ± 2.0 and 23.5 ± 2.1 nm, respectively. The hydrodynamic particle size of the gene-loaded micelles is understandably larger than the blank micelles, probably due to the incorporation of large and bulky biomolecules within the core. Moreover, these as-prepared RGD@P123@AP-2α nanocomposites can be easily dispersed in water to form a stable dispersion, and is very stable within almost three months.

**Table 1:** Values of different copolymer sizes loaded with RGD or AP-2α stored at 4 °C for different time intervals, respectively (*n* = 16).

Group	Original Size	Stored at 30 Days	Stored at 90 Days
P123	19.3 ± 1.5 nm	20.1 ± 2.1 nm	21.4 ± 2.3 nm
P123@AP-2α	21.2 ± 2.0 nm	22.5 ± 3.1 nm	23.3 ± 2.5 nm
RGD@P123@AP-2α	23.5 ± 2.1 nm	23.8 ± 2.3 nm	25.3 ± 3.2 nm

### 2.2. In Vitro Release of AP-2α Expression Plasmids from RGD@P123 Nano composites 

**Figure 2:**
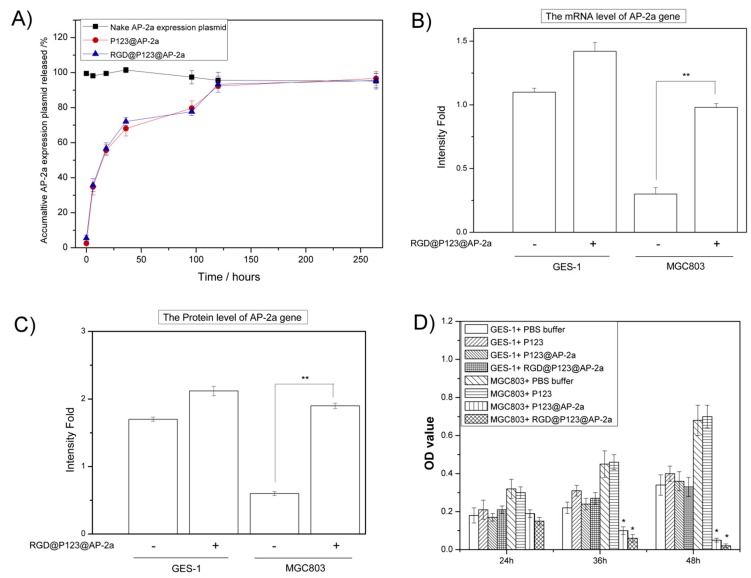
(**A**) Control release profiles of Activating protein 2α family (AP-2α) expression plasmids form RGD modified P123 nanoparticles; (**B**) mRNA and (**C**) protein expression level of *AP-2α* gene in GES-1 and MGC803 cells after incubation with RGD@P123@AP-2α nanocomposites; (**D**) MTT analysis of GES-1 and MGC803 cells after incubation with phosphate buffer solution (PBS), P123@AP-2α and RGD@P123@AP-2α nanocomposites, respectively. * *p* < 0.05, ** *p* < 0.01.

Figure 2A showed that the *in vitro* release ratio of both P123@AP-2α and RGD@P123@AP-2α nanocomposites is similar, but quicker than that of the naked AP-2α expression plasmid, which may result from “relaxation switch” effect [25]. Furthermore, both P123@AP-2α and RGD@P123@AP-2α nanocomposites exhibited a substantial burst release during the first 36 h incubation in phosphate buffer solution (PBS) buffered saline. The accumulative ratio of AP-2α expression plasmids released from P123 and RGD@P123 nanocomposites is 65.1% ± 3.1% and 72.1% ± 2.3% after incubation for 36 h. It was proposed that a slow release of AP-2α expression plasmids in either P123 or RGD@P123 nanocomposites could sustainably release after a burst phase over 11 days compared with naked AP-2α expression plasmids. It has been shown that P123 present on the surface of nanoparticles may function as a steric barrier that limits diffusion and release of AP-2α expression plasmids from the nanoparticles, suggesting P123 nanoparticles could provide a long period of releasing AP-2α expression plasmids in the body. Additionally, the encapsulation efficiency (*ee*) value of our prepared P123 naoncomposites is 67.4% ± 2.1%.

The biological activity of AP-2α expression plasmids is the key point for following research, especially after encapsulation into our prepared copolymers nanoparticles. Thus, the AP-2α mRNA and protein level in GES-1 cells and MGC803 cells after incubating with RGD@P123@AP-2α nanocomposites were analyzed by RT-PCR and western-blotting. As shown in Figure 2B,C, both mRNA and protein levels of AP-2α in MGC803 cells were significantly up-regulated after treatment with RGD@P123@AP-2α nanocomposites compared those in the blank group, while GES-1cells also exhibited a similar increase of AP-2α expression after RGD@P123@AP-2α nanocomposites treatment.

### 2.3. The Biocompatibility and Antitumor Activity of RGD@P123@AP-2α Nano composites 

To further determine whether RGD@P123@AP-2α nanocomposites were suitable for gene therapy in the biological environment, we used MTT analysis to evaluate their cytotoxicity *in vitro*. MTT analysis data in Figure 2D shows that the cell viability of 293T cells cultured with RGD@P123@AP-2α nanocomposites was similar to blank group within both 24, 36 and 48 h. It is suggested that RGD@P123@AP-2α nanoclusters only cause minor cytotoxicity for normal cells, in accordance with previous published results that both coploymers nanoparticles possess good biocompatibility. However, there is significant inhibition of gastric cancer MGC803 cells’ proliferation after co-culturing with RGD@P123@AP-2α for 36 and 48 h (shown in Figure 2D). This can be concluded that our prepared RGD@P123@AP-2α causes antitumor activity by up-regulating AP-2α protein expression in gastric cancer cells. Meanwhile, P123@AP-2α group also exhibited a toxic effect for MGC803 cells, which could be ascribed to the non-specific interaction with cellular receptors after a long incubation time.

**Figure 3:**
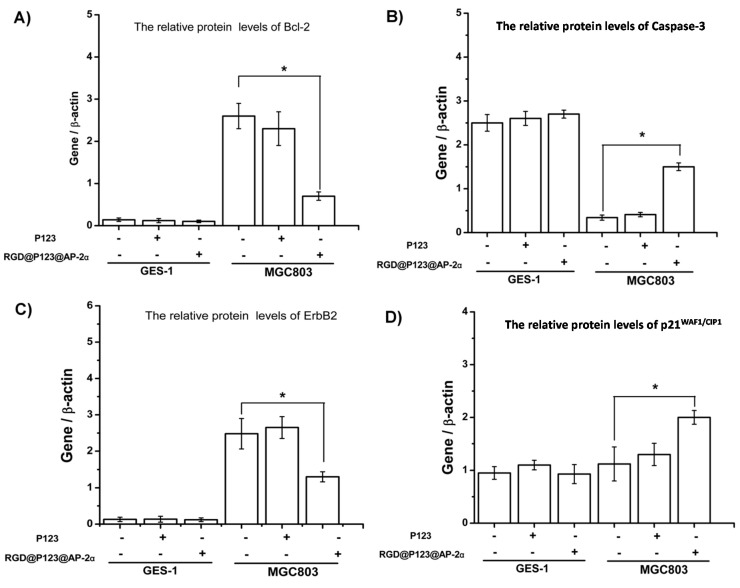
Western blotting analysis the changes of and other gastric cancer-associated proteins including (**A**) Bcl-2; (**B**) Caspase-3; (**C**) ErbB2; and (**D**) p21^WAF1/CIP1^ in GES-1 and MGC803 after incubation with RGD@P123@AP-2α nanocomposites, respectively. * *p* < 0.05.

Expression of some related proteins in the tumor tissue can reflect the potential mechanism of AP-2α in gene therapy. As shown in Figure 3, Bcl-2, caspase-3, ErbB2 and p21^WAF1/CIP1^ protein expression level in the primary gastric cancer model were determined by Western blotting (Figure 3). The result showed that the up-regulation of AP-2α expression could inhibit the protein expression level of Bcl-2 and ErbB2, but increase the protein expression level of caspase-3 and p21^WAF1/CIP1^.

### 2.4. AP-2α Expression Attenuated Gastric Tumor Growth in Nude Mice

**Figure 4:**
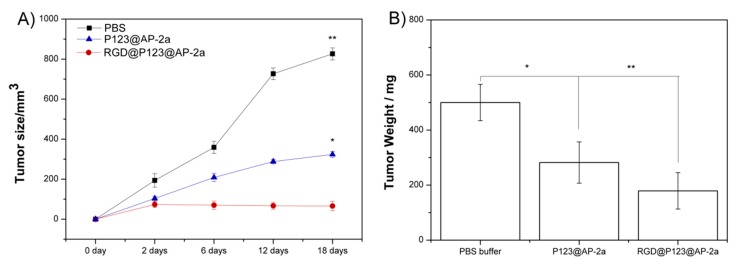
Detection of gastric tumor (**A**) size and (**B**) weight of nude mice under treating with PBS buffer, P123 and RGD@P123@AP-2α nanocomposites, respectively. * *p* < 0.05, ** *p* < 0.01.

A primary gastric tumor animal model was established in order to access the *in vivo* antitumor activity of our prepared RGD@P123@AP-2α nanocomposites. As shown in Figure 4A, the tumor growth curve of both P123@AP-2α and RGD@P123@AP-2α presented a smaller final tumor size than that in the PBS group. Notably, tumors treated with RGD@P123@AP-2α nanocomposites showed significantly reduced final tumor weights with a 64.8% lose than those in the control group (Figure 4B). Moreover, owing to their nanoscale size (shown in Table 1), the P123@AP-2α group also exhibited an antitumor activity by the enhanced permeability and retention (EPR) effect.

## 3. Experimental Section

### 3.1. The Preparation of Carboxyl-Functionalized Pluronic P123 Copolymer Filled with AP-2α Expression Plasmid

The AP-2α protein expression plasmid (pCMV6-AP-2α) was constructed in our lab. The AP-2αCDS region was amplified by polymerase chain reaction with KOD-Plus-Ver.2 (TOYOBO Co., Ltd., Osaka, Japan) according to the manufacturer’s protocol. The sequence of the cloned AP-2α CDS region was confirmed by DNA sequencing.

P123 nano micelles were prepared using the thin-film hydration method as described previously [24]. Briefly, the carboxyl-functionalized Pluronic P123 copolymer was prepared using a reaction mixture containing nitrile-functionalized copolymer Pluronic P123, NaOH, and ultrapure water in a round bottomed flask, the mixture was pre-stirred for 2 h in order to make a well-mixed phase and a pre-reaction, then the mixture was heated to 105 °C for 7 h under a congealed refluxing condition. After cooling, the mixture was extracted by dichloromethane and the organic phase was dehydrated over excessive anhydrous Na_2_SO_4_ and concentrated by rotary evaporation. The crude product was washed several times with ligarine (bp. 35–60 °C). The resulting product was dried in a vacuum oven at 25 °C for 3 days. The resulting activated micelles were covalently linked to RGD cycle pipetides (1% weight compared with polymer concentration) via 1-ethyl-3-[3-dimethylaminopropyl] carbodiimide hydrochloride (EDC) mediated coupling reaction. The peptide sequence is cycle (Arg-Gly-Asp-d-Phe-Lys) (c(RGDfK)). Then, 100 mM AP-2α protein expression plasmid, and 50 mM RGD-functionalized copolymer Pluronic P123 were dissolved in methyl cyanides under the help of ultrasonic treatment, and dried in a rotary evaporator at reduced pressure to form a thin-film layer, which was re-suspended in 1 mL ultrapure water containing 5% trehalose until completely hydrated.

The encapsulation efficiency (*ee*) of AP-2α protein on P123 copolymer was calculated by this equation: *ee* = (OD260 of pre-encapsulation − OD260 of post-encapsulation)/OD260 of pre-encapsulation. These as-prepared productions were characterized by SEM, TEM, NMR and DLS.

### 3.2. Cells and Cell Culture

The SV40-transformed immortal gastric epithelial cell GES-1 were preserved in our institute and maintained as recommended. The gastric cancer cell line MGC803 was obtained from the Chinese Academy of Sciences Cell Bank of Type Culture Collection and kept in our laboratory. All cell lines were cultured in Dulbecco’s Modified Eagle Medium (DMEM) supplemented with 10% fetal calf serum (FCS) (Sigma Chemical, St. Louis, MO, USA), 100 units/mL penicillin and 0.1 mg/mL streptomycin. The cells were maintained at 37 °C in a humidified chamber with 5% CO_2_.

### 3.3. In Vitro Release of AP-2α Protein Expression Plasmids from Nanoclusters

These as-prepared productions were incubated at 37 °C in PBS buffer with adding lysosomes at pH 4.7, respectively. Release of AP-2α protein expression plasmids was monitored at several time intervals including 0, 6, 18, 36, 96, 120 and 264 h. At each sampling time, the coploymers suspension was centrifuged for 15 min at maximum speeds. The supernatant was removed for determination of siRNA content, and an equal volume of PBS was replaced for continued monitoring of AP-2α plasmids release. The content of DNA in the aqueous solution was measured by Quant-iT™ Picogreen™ assay according to the manufacturer’s instructions (Invitrogen, Waltham, MA, USA).

### 3.4. MTT Analysis

A modified MTT (3-(4,5-dimethylthiazol-2-yl)-2,5-diphenyltetrazoliumbromide) test, in which the yellow MTT is reduced to a purple formazan by mitochondrial dehydrogenase in cells, was normally used to assess the cytotoxicity of nanomaterials. After 24, 36 and 48 h incubation with PBS buffer, P123, P123@AP-2α and RGD@P123@AP-2α nanocomposites, the viability of GES-1 cells and MGC803 cells were assessed, respectively. Briefly, GES-1 and MGC803 cells were washed three times with culture medium. The culture medium in each well of the plate was added 100 μL MTT (5 mg/mL in PBS). After 4 h incubation at 37 °C, the reaction solution was carefully removed from each well and 200 μL dimethyl sulfoxide was added. The plates were gently agitated until the formazan precipitate was dissolved, followed by measurement of OD values by spectrophotometry at 490 nm with an ElX-800 Microelisa reader (Bio-Tek Inc., Winooski, VT, USA).

### 3.5. In Vitro Evaluation of RGD@P123@ AP-2α Nanocomposites

In a typical experiment, both GES-1 and MGC803 cells were treated with and without RGD@P123@AP-2α nanocomposites. Total cellular RNA was isolated using the extraction kit (Qiagen Co., Hilden, Germany), according to the manufacturer’s instructions. The total RNA was subjected to reverse transcription with an oligo-dT primer to obtain cDNA. Primers were designed and used according to our previous works [26,27]. After extracting total cellular protein by repeating the same step, the protein level of related gene *Bcl-2*, *caspase-3*, *ErbB2*, *p21^WAF1/CIP1^* and reference gene *β-actin* in cells were analyzed by Real-time PCR (RT-PCR) and Western blotting, respectively. *β-Actin* was used as the normalized control.

### 3.6. Establishment of Animal Models

All animal studies complied with current ethical considerations with the approval of the Institutional Animal Care and Use Committee of Shanghai. Mice were obtained from the Experimental Animal center of Chinese Academy of Sciences, Shanghai, China. Female BALB/c mice 4–6 weeks old were subcutaneously injected in the right side of the back with 1 × 10^6^ MGC803 cells/mouse. PBS buffer, P123@AP-2α and RGD@P123@AP-2α nanocomposites were tail vein-injected into mice. Tumor volumes were measured every 6 days for tumor growth post-injection and recorded at each measured point. Mice were killed 18 days after injection of cells and tumor weights were recorded. Tumor growth inhibition rate was calculated as follows: percentage of tumor growth inhibition = (1 − *M*_T_/*M*_C_) × 100%, where *M*_T_ and *M*_C_ represent the mean tumor masses in treatment and control groups, respectively.

### 3.7. Statistical Analysis

All statistical analyses were carried out using the SPSS 17.0 statistical software package. Each experiment was performed independently at least twice with similar results. *p* < 0.05 in all cases was considered statistically significant.

## 4. Conclusions

In this study, we successfully prepared novel RGD@P123@AP-2α nanocomposites for gastric cancer gene therapy and evaluated its antitumor activity *in vitro* and *in vivo*. The characterization of freshly prepared RGD@P123@AP-2α nanocomposites exhibited low cytotoxicity, highly dispensability and good stability. Moreover, these as-prepared RGD@P123@AP-2α nanocomposites could encapsulate and protect AP-2α expression plasmids, and demonstrate a favorable gene release kinetic *in vitro*. These RGD@P123@AP-2α nanocomposites enable us to significantly inhibit gastric cancer cells growth *in vitro* and *in vivo* by up-regulating AP-2α protein expression. This not only provides a feasible nanomedicine for gastric cancer treatment, but also paves a novel pathway for treating other cancers in the future.
